# Measurement of lactate levels in postmortem brain, iPSCs, and animal models of schizophrenia

**DOI:** 10.1038/s41598-019-41572-9

**Published:** 2019-03-25

**Authors:** Courtney R. Sullivan, Catharine A. Mielnik, Adam Funk, Sinead M. O’Donovan, Eduard Bentea, Mikhail Pletnikov, Amy J. Ramsey, Zhexing Wen, Laura M. Rowland, Robert E. McCullumsmith

**Affiliations:** 1 Taconic Biosciences, Rensselaer, NY USA; 2 Department of Pharmacology and Toxicology, University of Toronto, Toronto Ontario M5S, 1A8, Toronto, Canada; 3 Department of Neurosciences, University of Toledo, Toledo, OH 43614 USA; 4 Center for Neurosciences (C4N), Department of Pharmaceutical Biotechnology and Molecular Biology, Vrije Universiteit Brussel, Brussels, Belgium; 5 Department of Psychiatry and Behavioral Sciences, Johns Hopkins Medicine, Baltimore, Maryland 21287 USA; 6 Department of Psychiatry and Behavioral Sciences, Emory University, Atlanta, Georgia 30322 USA; 7 Department of Psychiatry, University of Maryland School of Medicine, Baltimore, Maryland 21201 USA

## Abstract

Converging evidence suggests bioenergetic defects contribute to the pathophysiology of schizophrenia and may underlie cognitive dysfunction. The transport and metabolism of lactate energetically couples astrocytes and neurons and supports brain bioenergetics. We examined the concentration of lactate in postmortem brain (dorsolateral prefrontal cortex) in subjects with schizophrenia, in two animal models of schizophrenia, the GluN1 knockdown mouse model and mutant disrupted in schizophrenia 1 (DISC1) mouse model, as well as inducible pluripotent stem cells (iPSCs) from a schizophrenia subject with the DISC1 mutation. We found increased lactate in the dorsolateral prefrontal cortex (p = 0.043, n = 16/group) in schizophrenia, as well as in frontal cortical neurons differentiated from a subject with schizophrenia with the DISC1 mutation (p = 0.032). We also found a decrease in lactate in mice with induced expression of mutant human DISC1 specifically in astrocytes (p = 0.049). These results build upon the body of evidence supporting bioenergetic dysfunction in schizophrenia, and suggests changes in lactate are a key feature of this often devastating severe mental illness.

## Introduction

Schizophrenia is a devastating illness that affects 1% of the general population and displays a wide range of psychotic symptoms, as well as cognitive deficits and profound negative symptoms that are often treatment resistant^1–5^. Cognition is intimately related to synaptic function, which relies on the ability of cells to generate bioenergetic substrates. For example, glutamate released at the synapse signals increased energetic demand to astrocytes and enhances production of bioenergetic substrates such as lactate^6–8^. Lactate is then transported out of astrocytes and into neurons through monocarboxylate transporters (MCTs) where it can be utilized to produce energy in the tricarboxylic acid (TCA) cycle^9,10^. This mechanism, called the “astrocyte-neuron lactate shuttle,” uses lactate to support neuronal oxidative phosphorylation even in aerobic conditions when energy demand is high and neurons are unable to effectively upregulate glycolysis (and thus TCA cycle substrates)^11–14^. Supporting the hypothesis that lactate is needed for cognition, learning tasks in rats are accompanied with increases in extracellular lactate in the hippocampus, while perturbing the generation of lactate or its import into neurons produces amnesia^14^. The effect of impaired lactate generation is rescued when exogenous lactate is applied, suggesting that the lactate shuttle is necessary for long-term memory and synaptic activity^14^. Thus, lactate and connected bioenergetic pathways warrant further investigation in disorders of cognition, including schizophrenia.

There are several lines of evidence suggesting that the supply and transport of lactate in living schizophrenia patients is disrupted. Previous studies reported elevated lactate concentrations in both cerebral spinal fluid (CSF) and stimulated peripheral blood mononuclear cells (PBMCs) obtained from schizophrenia patients^15,16^. In the same PBMC study, there was also a significant increase in the expression of lactate dehydrogenase B (LDHB) and glucose-6-phosphate isomerase (GPI) protein in stimulated and unstimulated PBMCs in first-onset antipsychotic-naïve schizophrenia subjects^16^. LDH and GPI are glycolytic enzymes necessary for the metabolism of glucose into bioenergetic substrates. These findings support the hypothesis that biological process involved in the production of lactate are abnormal in schizophrenia, possibly contributing to the cognitive deficits found in this illness.

*In vivo* proton magnetic resonance spectroscopy (MRS) studies offer a noninvasive approach to directly study brain bioenergetics in schizophrenia. Interestingly, a study employing high field (7T) MRS demonstrated elevated *in vivo* brain lactate levels in patients with schizophrenia, possibly indicating metabolic dysfunction with a shift towards anaerobic glycolysis^17^. These elevations in lactate were associated with lower general cognitive function and functional capacity in the total sample, including visual learning, processing speed, and reasoning/problem solving cognitive domains (n = 31/group)^17^. Supporting these findings, high-resolution magic angle spinning (HRMAS) ^1^H NMR spectroscopy-based metabolomics analysis revealed a 1.5 fold change (FC) increase in lactate levels in prefrontal cortex white matter in schizophrenia^18^. Similarly, postmortem studies have reported elevated lactate levels in the striatum and cerebellum in schizophrenia subjects versus controls^19,20^. Despite these findings, there is some controversy regarding whether or not changes in lactate are part of a pathological mechanism related to the illness, or altogether secondary to the effects of medication or other postmortem factors that may impact lactate levels. To address this controversy in the field, we investigated lactate levels in postmortem schizophrenia samples from the dorsolateral prefrontal cortex (DLPFC) (Table 1). We supplemented these studies by measuring lactate in two animal models of schizophrenia and inducible pluripotent stem cells (iPSCs) from patients, allowing us to control for the effects of antipsychotic medication, postmortem interval, and other factors that limit interpretation of postmortem brain studies.

**Table 1:** Subjects table.

	CTL	SCZ
N	16	16
Sex	12 m,4 f	13 m,3 f
pH	6.6 ± 0.2	6.6 ± 0.3
PMI	12 ± 5	13 ± 6
Age	43 ± 9	45 ± 11
Rx	16/0/0	2/11/3

Subject demographics. Control subjects (CTL), schizophrenia (SCZ), postmortem interval (PMI), male (m), female (f), off or unknown/on typical/atypical antipsychotic treatment (Rx). Data are mean ± SEM.

## Results

**Figure 1:**
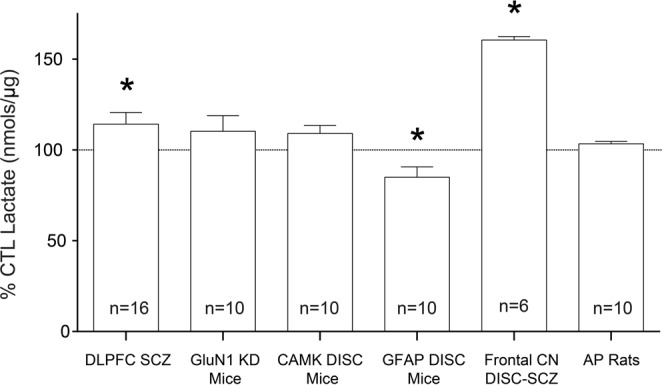
Lactate levels expressed as %CTL (or wildtype). Lactate concentration (nmoles/µg protein) measured in the dorsolateral prefrontal cortex (DLPFC) of control subjects (CTL) and subjects with schizophrenia (SCZ) expressed as % CTL. Lactate concentration was also measured in frontal cortex tissue homogenate from GluN1 knockdown (KD) mice (versus wildtype), the mutant disrupted in schizophrenia 1 (DISC1) mouse model with inducible expression of mutant human DISC1 in GFAP or CAMK positive cells (versus wildtype), cell lysates of inducible pluripotent stem cells from a schizophrenia patient (n = 6 technical replicates) with a mutation in the DISC1 gene differentiated into frontal cortical neurons (CN) (versus unaffected family member), and prefrontal cortex homogenate from rats treated with the antipsychotic (AP) haloperidol-deaconate (28.5 mg/kg q 3 weeks) (versus vehicle) for 9 months. Data are expressed as mean ± SEM. *P < 0.05.

**Figure 2:**
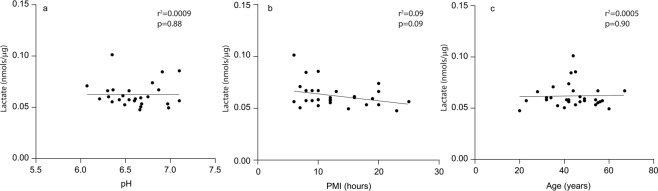
Correlation analyses. Bivariate plots (linear regression) of lactate concentration (nmoles/µg protein) versus pH (**a**), postmortem interval (PMI) (**b**), or age (**c**) in dorsolateral prefrontal cortex (DLPFC) in all control (CTL) subjects and subjects with schizophrenia (SCZ). Outliers removed with ROUT method (Q = 5%).

We detected an increase (12%) in lactate in subjects with schizophrenia in the DLPFC (p = 0.0433) (Fig. 1). Postmortem lactate levels were not associated with postmortem interval (PMI), pH, or age (Fig. 2).

**Figure 3:**
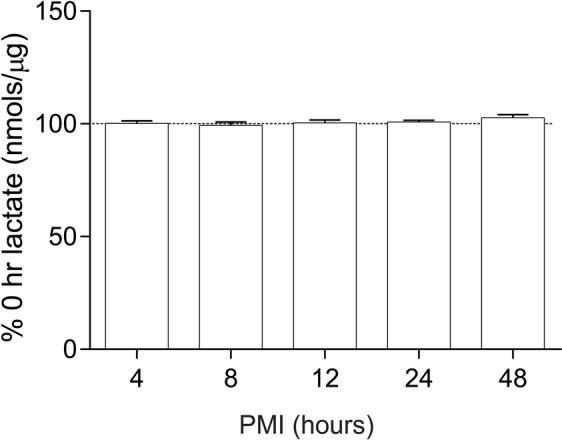
Lactate levels in rats simulating increasing PMIs. Lactate concentration (nmoles/µg protein) in the frontal cortex of rats simulating varying postmortem intervals (PMIs) expressed as percent of 0 hour PMI. Data are expressed as mean ± SEM (n = 3 per group). *P < 0.05.

We did not find any changes in lactate levels in rats treated with haloperidol-decanoate for 9 months (p = 0.065) or in rats with simulated PMIs up to 48 hours (p = 0.44) (Figs 1 and 3).

In the mutant GFAP DISC1 mouse model, we found a decrease (15%) in lactate in the frontal cortex (p = 0.049) (Fig. 1). We did not detect any changes in lactate in the mutant CAMKII DISC1 mouse model (p = 0.19) (Fig. 1). We did not find any changes in lactate in the frontal cortex of GluN1 KD mice when compared to WT littermate controls (Fig. 1).

In cell lysates from frontal cortical neurons differentiated from iPSCs from a schizophrenia patient, we found a significant increase (37%) in lactate compared to frontal cortical neurons differentiated from an unaffected family member (p < 0.0001) (Fig. 1).

## Discussion

Questions have been raised about the involvement of the lactate system in the bioenergetic pathophysiology of schizophrenia. Our findings of elevated lactate levels in the DLPFC builds upon current evidence of increased lactate levels in schizophrenia^17–20^. Previous hypotheses have suggested that alterations in lactate levels found in postmortem brain in schizophrenia could be secondary to antipsychotic treatments, long PMIs, or reduced pH. For instance, brain pH is dependent on the balance between acid-base transport (such as lactic acid) and is often negatively correlated with lactate levels^21,22^. We found no significant correlations between lactate levels in the DLPFC of schizophrenia patients and age, pH, or PMI (Fig. 2). Studies in living patients, which do not have confounds of PMI, have also demonstrated increased lactate levels in the prefrontal cortex, suggesting that increased lactate levels in the schizophrenia prefrontal cortex are not postmortem artifacts or a result of decreased pH^17^. However, there is evidence suggesting low pH is associated with increases in lactate in brain homogenate in animal models of psychiatric disorders, suggesting that changes in pH and lactate are part of the pathophysiology of severe mental illness^21^. This is supported by meta analyses of 10 existing postmortem pH datasets^21^. While our data do not support a direct link between brain pH and tissue lactate levels, accumulating evidence suggests changes in pH and lactate in severe mental illness are not artifacts of postmortem brain processing or medication treatment.

Although the elevation of lactate levels in schizophrenia suggests a dysregulation of bioenergetic processes, it may be interpreted in various ways. Elevated lactate levels in postmortem brain could result from the inability of neurons to take in and utilize bioenergetic substrates. For instance, if neurons were unable to produce adequate energy through glycolysis and oxidative phosphorylation, then a compensatory shift away from aerobic glycolysis in astrocytes when energy demand is high may result in the generation of higher amounts of lactate. The results presented here serve as indirect evidence of abnormalities of biochemical pathways involved in lactate production, and need to be confirmed by more sophisticated approaches such as heavy carbon labeling or Seahorse-based analyses in model systems.

There is also postmortem evidence for abnormally regulated glycolytic pathways and lactate shuttle components in schizophrenia. We have previously reported decreases in hexokinase (HK) and phosphofructokinase (PFK) activity in the DLPFC in schizophrenia, which could result in the abnormal production of bioenergetic substrates such as lactate^23^. Additionally, both elevated CSF lactate levels and MRS lactate peaks are generally considered a clinically relevant indicator for mitochondrial disease^24,25^. Lactate as an indicator of mitochondrial dysfunction (and thus abnormal bioenergetics) has implications for defects in processes like neurotransmission and synaptic plasticity, and could contribute to cognitive impairment in schizophrenia. There is evidence that many aspects of mitochondrial function are disrupted in schizophrenia, and it would be of interest to examine the expression and activity levels of mitochondrial markers in our schizophrenia cohort and other models^26^.

We also report abnormalities in lactate levels in an animal model of schizophrenia, the GFAP DISC1 mouse, and frontal cortical neurons differentiated from iPSCs of a patient with the DISC1 mutation (Fig. 1). Abnormal lactate levels in these cell-subtype specific models suggest that bioenergetic abnormalities in schizophrenia may be important for astrocyte and neuron crosstalk. Although these findings need to be replicated, this idea is supported by other cell-level bioenergetic findings that were not detected at the brain region-level^23^. A recent study in mice examining the effects of knocking down endogenous DISC1 on the metabolic function of astrocytes suggests DISC1 may be involved in the regulation of lactate production and shuttling when energy demand is high^27^. Primary astrocytes cultured from DISC1 mutant mice show abnormalities in the expression of GLUT1, LDHA, LDHB, and other glycolytic enzymes^27^. Changes in lactate levels in the hippocampus of DISC1 mutants were not accompanied by serum changes, suggesting a reduced production of lactate by brain astrocytes rather than from global changes in lactate metabolism^27^. Interestingly, systemic lactate treatment rescued the abnormal behaviors (including memory defects) associated with the DISC1 mutant mice^27^.

We did not detect any changes in lactate in our mutant CAMKII DISC1 mouse model (p = 0.19) (Fig. 1). These mice have a depletion of endogenous DISC1 in neurons as opposed to GFAP positive astrocytes^28^. This suggests that depletion of DISC1 in neurons is likely insufficient to lead to dysregulation of lactate production and transport, a process that is predominantly found in astrocytes and not neurons. If astrocytes are unable to upregulate glycolysis when energy demand is high, less lactate would be produced for the transport and use in neurons in these mice. The lack of detectable changes in lactate following a decrease in DISC1 in neurons could be due to normal astrocytes having the ability to bioenergetically compensate for the mutant neurons in the CAMKII DISC1 mice.

The present study has several limitations. First, there are limited demographical data available for our postmortem samples, and complete patient history (including information on the duration of diagnosis, disorder subtype, ect.) might be useful. Additionally, most of the patients in our studies were taking antipsychotic medication at time of death (Table 1). One previous study hypothesized that increases in lactate in schizophrenia is secondary to prior antipsychotic treatment rather than evidence for primary metabolic abnormalities^19^. They found that rats treated with haloperidol (n = 5, 0.8 mg/kg/day) and clozapine (n = 5, 5 mg/kg/day) for 4 weeks had an increase in lactic acid in the frontal cortex relative to vehicle (n = 5)^19,21^. However, the same study reported that lactate did not correlate with any measure of antipsychotic treatment in patients^19^. Moreover, we did not detect any differences in rats (n = 10) treated with haloperidol-decanoate for 9 months, suggesting that chronic antipsychotic treatment in rats does not increase lactate (Fig. 1). It is possible that antipsychotic medication leads to transient increases in lactate but is stabilized over time. It is also possible that lactate levels in antipsychotic treated rats are dose dependent, and that different dosing regimens of haloperidol could results in changes in the lactate system. Another limitation of this study is that no *in vivo* measurements were obtained from our animal models or human subjects. In addition, our pilot PMI animal studies have n = 3 animals per group and could benefit from additional animals and/or replication. Similarly, our frontal cortical neuron findings would be strengthened by including IPSCs from more schizophrenia patients and unaffected siblings. Finally, this study could also benefit from expanding the scope to include other critical bioenergetic pathways. Mitochondrial dysfunction has been linked to the pathogenesis of schizophrenia and key mitochondrial markers such as cytochrome c oxidase or other mitochondrial enzymes should be examined.

In summary, we report increases in lactate in the DLPFC in schizophrenia, as well as abnormal lactate levels in the frontal cortex of mutant GFAP DISC1 mice and frontal cortical neurons from a schizophrenia patient. We also found that these changes do not appear to be due to pH, age, or PMI effects, or due to treatment with antipsychotic drugs. Of note, our animal models of schizophrenia did not recapitulate the changes we found in postmortem brain or patient IPSCs, suggesting that these changes are likely a result of divergent mechanisms between schizophrenia and its models. For instance, the changes we observe in the GFAP DISC mice are likely driven by the well characterized mitochondrial defects found in DISC mutants, while the changes in schizophrenia are more subtle and likely secondary to a lifetime of synaptic dysfunction^28,29^. Supporting this notion, studies investigating the genetic risk for schizophrenia converge on genes/proteins associated with excitatory neurotransmission^30^ and functional deficiencies of DISC1 are associated with a number of secondary mitochondrial dysfunctions (decreased NADH dehydrogenase activities and reduced ATP contents)^31^. There is also data to support the hypothesis that developing a brain with broken synapses yields bioenergetic defects in adult animals^27,29,32^. Finally, while most of the genetic risk for schizophrenia is associated with synapse biology, some genomic risk alleles involve mitochondria and some metabolic factors, which may also contribute to the pathogenesis of schizophrenia^33,34^.

Targeting the lactate system by modulating lactate or glycolytic pathways could balance or bypass disruptions in bioenergetic function. For example, MCTs also transport other monocarboxylates such as ketone bodies, which can be used as an energy source for the brain^35^. Interestingly, a ketogenic diet normalized pathological behaviors in the MK-801 NMDA hypofunction model of schizophrenia^36^. Further, impaired prepulse inhibition (PPI) of the startle response (a translationally validated endophenotype of schizophrenia) in the MK-801 NMDA hypofunction model was prevented following ketogenic diet^37^. There have also been case studies suggesting ketogenic diet may be an effective treatment for positive and negative symptoms in schizophrenia and schizoaffective disorder, as well as metabolic dysfunction^38,39^. In conclusion, accumulating evidence suggests increases in lactate could be a pathological indicator of bioenergetic defects in cognitive disorders including schizophrenia.

## Materials and Methods

### Tissue acquisition and preparation

Dorsolateral prefrontal cortex (DLPFC, Brodmann area 9) postmortem brain samples originated from the Maryland Brain Collection and were distributed by both the Maryland Brain Collection and the Alabama Brain Collection (ABC). All methods were carried out in accordance with relevant guidelines and regulations, and all experimental protocols were approved by the University of Cincinnati. Human postmortem studies in this manuscript are IRB exempt (exemption 4). The cohort consisted of subjects with schizophrenia (n = 16) and nonpsychiatrically ill comparison subjects (n = 16) (Table 1). Subjects were diagnosed with schizophrenia based on DSM-IV criteria. The medical records of the subjects were examined using a formal blinded medical chart review instrument, as well as in person interviews with the subjects and/or their caregivers, and permissions were obtained from next of kin as previously described^40^. Schizophrenia and comparison groups were matched for sex, age, pH, and PMI (Table 1).

### Postmortem lactate assays

Two 14 µm sections per subject were scraped from glass slides and samples resuspended in 30–50 µl cold 1x PBS buffer with HALT protease and phosphatase inhibitor and then centrifuged at max speed in a Sorvall Legend Micro 21 R centrifuge for 4 minutes at 4 °C. The supernatant was collected, assayed for protein content, and evaluated using the manufacturer’s protocol (L-Lactate Assay kit colorimetric, ab65331, Abcam, Cambridge, Massachusetts, USA) with the following changes. Samples (0.5 µg total protein per well) were heated at 95 °C for 5 minutes prior to the assay to inactivate any endogenous enzymes. Each sample was assayed with and without enzyme to control for any remaining endogenous enzyme activity. Samples were incubated with reaction mix for 30 minutes at room temperature and then read at the 450 nm using a BioTek ELx800. Data are presented as sample lactate concentration (nmol/µg) with enzyme minus concentration without enzyme. Assays had a coefficient of variability of 1–3%.

### Antipsychotic and postmortem interval rodent studies

Rodent studies were performed in accordance with the IACUC guidelines at the University of Alabama-Birmingham. Additionally, all experimental protocols were approved by the University of Alabama. Sample size was chosen to minimize the number of animals necessary to be sufficiently powered based on a priori power calculations from previous studies^41^. Animal groups were alternated in terms sacrifice. Investigators were blinded until after data collection. Adult male Sprague–Dawley rats (250 g) were housed in pairs and maintained on a 12-h light/dark cycle. Dissection of rodent brains included removal of olfactory bulb and sectioning tissue from the most rostral point of the prefrontal cortex, the frontal pole.

For antipsychotic studies, rats received 28.5 mg kg−1 haloperidol-decanoate or vehicle (sesame oil) by intramuscular injection every 3 weeks for 9 months. We used haloperidol, a typical antipsychotic, for these studies because the majority of our schizophrenia subjects (11/16) were taking typical antipsychotics at time of death. Brain tissue lactate concentrations were assayed in the frontal cortex (n = 10 per group) following the 9 month treatment period.

For postmortem interval studies, PMIs were simulated by keeping rat brain tissue at room temperature for time points of 0, 4, 8, 12, 24, and 48 hours (n = 3 per time point) prior to dissection.

### Antipsychotic and PMI rat lactate assay

Lactate levels were assayed in the frontal cortex of the rat brains simulating varying PMIs as well as rats treated with haloperidol-decanoate. For both studies, 14 µm sections per subject were scraped from glass slides and samples resuspended in 30 µl cold 1x PBS buffer with HALT protease and phosphatase inhibitor. Once resuspended, samples were centrifuged at 13,000 g in a Sorvall Legend Micro 21 R centrifuge for 10 minutes at 4 °C. The supernatant was collected, assayed for protein content (using the bicinchoninic acid method, as described first by^42^) and evaluated using the manufacturer’s protocol (L-Lactate Assay kit colorimetric, ab65331, Abcam, Cambridge, Massachusetts, USA) with the following changes: Samples (2 µg (PMI study) or 1 µg (antipsychotic study) total protein per well) were heated at 95 °C for 5 minutes prior to the assay to inactivate any endogenous enzymes. Each sample was assayed with and without enzyme to control for any remaining endogenous enzyme activity (sample lactate concentration with enzyme minus concentration without enzyme). Samples were incubated with reaction mix for 30 minutes at room temperature and then read at the 450 nm using a BioTek ELx800. Lactate levels in the haloperidol treated rat group (Fig. 1) and PMI rat groups 4, 8, 12, 24, 48 hr (Fig. 3) are presented as a percent control. Assays had a coefficient of variability of 1–3%.

### DISC1 mice and GluN1 KD mice lactate assays

We have employed two mouse models of schizophrenia: the mutant DISC1 model with inducible expression of mutant human DISC1 in GFAP or CAMKII positive cells to down-regulate the level of endogenous DISC1 in astrocytes or neurons respectively (as previously described^28^) and the GluN1 KD model, a genetic knockdown of the GluN1 subunit of NMDA receptors resulting in hypofunction^43^. For the DISC1 model study, frontal pole was homogenized in 250 µl of cold homogenization buffer with HALT protease and phosphatase inhibitor. For the GluN1 KD study, two frontal cortex 14 µm sections per animal were scraped from glass slides and samples resuspended in 30 µl cold 1x PBS buffer with HALT protease and phosphatase inhibitor. Once resuspended, both DISC1 and GluN1 KD samples were centrifuged at 13,000 g in a Sorvall Legend Micro 21 R centrifuge for 10 minutes at 4 °C. The supernatant was collected, assayed for protein content (using the bicinchoninic acid method, as described first by^43^) and evaluated using the manufacturer’s protocol (L-Lactate Assay kit colorimetric, ab65331, Abcam, Cambridge, Massachusetts, USA) with the following changes: Samples (4 µg (DISC1) or 6 µg (GluN1 KD) total protein per well) were heated at 95 °C for 5 minutes prior to the assay to inactivate any endogenous enzymes. Each sample was assayed with and without enzyme to control for any remaining endogenous enzyme activity (sample lactate concentration with enzyme minus concentration without enzyme). Samples were incubated with reaction mix for 30 minutes at room temperature and then read at the 450 nm using a BioTek ELx800. Lactate data for both studies are presented as a percent control (Fig. 1). Assays had a coefficient of variability of 1–3%.

### Differentiated frontal cortical neuron lactate assays

Cell lysates of iPSCs from one schizophrenia patient with a mutation in the DISC1 gene and one nonpsychiatrically ill family member (demographics described in (5 2)) were assayed for protein content and evaluated using the manufacturer’s protocol (L-Lactate Assay kit colorimetric, ab65331, Abcam, Cambridge, Massachusetts, USA) with the following changes: Samples (4 µg total protein per well) were heated at 95 °C for 5 minutes prior to the assay to inactivate any endogenous enzymes. Each sample was assayed with and without enzyme to control for any remaining endogenous enzyme activity. Samples were incubated with reaction mix for 30 minutes at room temperature and then read at the 450 nm using a BioTek ELx800. Data are presented as sample lactate concentration with enzyme minus concentration without enzyme. Assays had a coefficient of variability of 1–3%.

### Statistical analysis

All outliers were removed using the ROUT method before statistical analysis and graphing (Q = 5%). All dependent measures were tested for normalcy and homogeneity of variance using D’Agostino & Pearson omnibus normality test. All data were normal, thus we performed Student’s t-tests. We probed for associations between our dependent measures and pH, PMI, and age using correlation analyses (linear regression).
